# Using Personas as a Tool for People-Centred Segmentation and Health Systems Planning in Ontario, Canada

**DOI:** 10.5334/ijic.9002

**Published:** 2026-05-21

**Authors:** Casey Chu, Kerry Kuluski, Arija Birze, Junhee Baek, Louis Wong, Catherine Donnelly, Brianne Wood, Jessica Logozzo, Laura Rosella

**Affiliations:** 1 Institute for Better Health, Trillium Health Partners, Mississauga, Ontario, Canada; 2 Dalla Lana School of Public Health, University of Toronto, Toronto, Ontario, Canada; 3 Health Services and Policy Research Institute, Queens University, Kingston, Ontario, Canada; 4 Dr. Gilles Arcand Centre for Health Equity, NOSM University Thunder Bay, Ontario, Canada; 5 Thunder Bay Regional Health Sciences Centre and Northwestern Ontario Hospitals, Thunder Bay, Ontario

**Keywords:** population health management, population segmentation, community engagement, qualitative methods, personas

## Abstract

**Introduction:**

Health systems are increasingly using population segmentation for planning to organise populations into segments with similar health characteristics. However, given that these data-driven approaches rely on available data, they fail to encompass important social determinants of health, thereby making decision-making challenging. Segmentation is powerful, but little is known about how missing information could be incorporated to more accurately characterise segments.

**Description:**

We demonstrate a process that uses personas [i.e., fictional stories] to apply the outputs of population segmentation in Ontario, Canada. First, we created personas using available data to represent individuals in a population segment. Second, we conducted focus groups with service users, caregivers, and providers to understand what information was missing from the persona. We conducted two iterative rounds of focus groups with 27 participants to test and assess this process on the frailty population segment.

**Results:**

Findings revealed the preferred design and types of information to present in a persona to represent individuals from a segment. Participants also identified what information was pertinent or missing to better understand the frailty population segment in Ontario.

**Conclusion:**

Personas can be useful for health system planners as a mechanism to incorporate community perspectives to enhance data-driven segments for planning.

## Introduction

A population health management approach to health system planning involves tailoring healthcare strategies to the range of healthcare needs in entire populations, not only those with specific illnesses or needs [1,2]. It also applies an integrated conception of health needs, encompassing multiple health, social, and environmental factors [3,4]. Health systems are increasingly using data-driven population health management tools to understand their populations better and develop tailored approaches to support optimal care delivery.

Population segmentation is an analytic approach that divides a population into groups with similar health characteristics and needs [5] to facilitate population health management. This approach has historically relied on routinely collected healthcare data [i.e., health administrative data] to stratify populations into mutually exclusive segments or groups of individuals according to health status and needs. The resulting groups allow health systems to identify and understand the needs of groups with different levels of risk and resource use [6], which can guide health systems in targeting resources and proactively organising and delivering care to each group. The data from which these segments are created offer robust health information but are limited in providing adequate information on the broader determinants of population health [e.g., individual needs and priorities]. As a result, population segments do not fully reflect the diverse needs and contexts of the people within them.

Segmentation has been useful at the small scale for health systems to identify and predict high-resource users [7,8], organise services [9], or target prevention activities to reduce emergency department [ED] visits [10]. On a larger scale, it has been used to roll out population-wide strategies such as vaccination [11] and diabetes programs [12]. An example of segmentation applied at the health systems level is the “Know Thy Patient” initiative at Parkland Health in Dallas, Texas, which segmented 630,289 people in their area to guide the design of clinical programs [13]. The initiative used administrative data to generate eight clusters and characterised each in a profile [also referred to as personas], which featured a fictional community member, their sociodemographic and health characteristics, residential area-level characteristics, and healthcare utilisation measures.

Personas are specific, concrete, and detailed descriptions of fictional target users of a system created using well-understood, highly specified data about real people [14,15]. Some have described personas as a “user modelling technique used to provide insight into the audience and communicate their needs and behaviour more convincingly and memorably,” and a way to represent a group of people to target empathy toward [17]. Using personas focuses on understanding “important users,” and some have argued that personas enable faster and more confident decision-making by allowing leaders to know who their audience is and what they need [17]. While Parkland Health’s personas use all relevant and available administrative data, they are limited in informing health programs as the data do not account for service user-reported needs and priorities. These information gaps will need to be gathered from other datasets and importantly, engaging communities within each segment to maximise the utility of segments as a tool for guiding integrated health systems planning and tailored program development.

Gaps in health administrative data limit population segmentation. Firstly, these data may be of poor quality, reflect past healthcare utilisation, and not explicitly consider known determinants of population health. This precludes the potential ability to address service users’ comprehensive needs and priorities, thus limiting the possibility of using segmentation results to guide people-centred care, which, “adopts individuals’, carers’, families’ and communities’ perspectives as participants in, and beneficiaries of, trusted health systems that are organised around the comprehensive needs of people rather than individual diseases, and respects social preferences” [18]. Consequently, results can potentially misrepresent and be oppressive towards certain populations [19]. Second, health administrative data are primarily physician-based and do not capture the utilisation of social or community programs and care offered by non-physician providers [i.e., allied health professionals, unregulated healthcare providers, family caregivers and volunteers] that play an essential role in addressing broader determinants of health. Without this information, input from individuals who use health and social care, their caregivers, and allied health and social care providers is essential. Incorporating this information into segments would provide a better understanding of population needs and could drive people-centred healthcare services – care that aligns with the needs and priorities of service users in the community. This paper aims to assess how personas, a method for generating profiles that represent the diversity of individuals within a population of interest, can be applied to enhance population segmentation. Specifically, we describe a case for how this approach was applied in Ontario, Canada, to incorporate community perspectives into making population segments more reflective of the communities they represent to support integrated health system planning.

### Applying Personas to Population Segmentation

Personas have been heavily utilised to describe user characteristics and improve user experiences in the education, industrial, and software industries [20,21,22,23]. More recently, it has been used in the health sciences to improve client and service user experience [17,24,25]. One local example from Northwestern Ontario, Canada, used personas co-designed with providers and care coordinators to design community-based health and social care packages for individuals waiting for long-term care [i.e., nursing home care] [26]. Personas can help contextualise data-centric population segments and incorporate community input regarding health needs and priorities.

This proof-of-concept study sought to understand and evaluate how personas can be used to better understand population segments. We engaged with service users, caregivers, and health and social care providers to gain a deeper understanding of a selected segment using personas. Working across Ontario, Canada, we selected and generated preliminary personas on the frailty population segment [i.e., those with frailty-related conditions]. Then, we conducted focus groups with service users, caregivers, and care providers, to highlight critical information related to sociodemographics, social wellbeing, and needs that were not captured in health and other administrative data. We also used these focus groups to assess our approach for applying personas to population segmentation for health systems planning.

## Description of Approach

### Setting and Context

This study took place in the province of Ontario, Canada. In 2019, the provincial government initiated the Ontario Health Teams [OHTs] health system model to integrate different healthcare providers within a community to deliver care seamlessly and collaboratively under one unified healthcare system. Each OHT is challenged with carrying out population health management, which involves taking “a population-health perspective to delivery of health and other human services in a [*people-centred*] manner” [27,28]. OHTs have begun to explore health administrative data on their respective population and sub-populations through tools such as segmentation [29]. In this project, we partnered with OHTs spanning across the province, including the Mississauga, Frontenac Lennox Addington, Nipissing, and the Northwestern Ontario Regional Integrated Care Working Group [comprising multiple OHTs in Northwestern Ontario] to collaboratively refine our methodology, recruit participants, and interpret findings. We focused on the frailty segment, a common priority population across OHTs [30]. These segments were extracted for the Mississauga OHT, which serves a diverse population of 859,392 people with a wide range of health issues [31].

### Design

**Figure 1:**
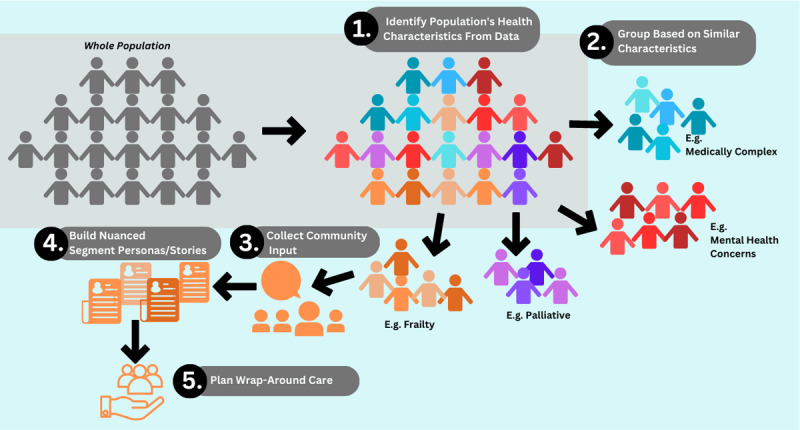
The conception of the people-centred segmentation approach.

This is a proof-of-concept study of a larger mixed-method project that aims to understand how to conduct people-centred segmentation at the health system level [full project depicted in Figure 1]. Specifically, we piloted a novel method for engaging with communities through focus groups to form personas representing population segments [steps 3 and 4 from Figure 1].

We started this pilot by identifying a priority population segment, which all of our partner OHTs were interested in characterising, and used available sociodemographic and health data to create preliminary segment personas. Then, we reviewed these preliminary personas through a first round of two virtual focus groups [one with service users and caregivers and a second with care providers from a local community [Mississauga, Ontario] to gather insights on each segment’s sociodemographics, social wellbeing, and broader health needs. Lastly, we analysed these focus group data qualitatively and revised our personas and focus group discussion processes. Lastly, we conducted a second round of two focus groups with service users, caregivers, and care providers across Ontario, applying the learnings from round one.

### Data Sources and Preliminary Persona Generation

As an existing population health management technique, personas are often created using qualitative data alone [32], allowing for a nuanced and detailed assessment of user motivations, expectations, and needs. However, because of the small sample size associated with qualitative methods, there is no way to determine the proportion of the user population that each persona represents, and unique characteristics may be inadvertently omitted, or outliers may be overrepresented. The addition of quantitative data, forming data-driven personas, addresses many of these concerns [17,33], with many experts advocating for mixed methods approaches to persona creation [34].

#### Data Sources

We used population segments already generated using health administrative data by an Ontario primary care research group, the Innovations Strengthening Primary Healthcare Through Research [INSPIRE-PHC] from March 31, 2020 [35] for each OHT and publicly available on the ontariohealthprofiles.ca website [36]. The databases that were used by INSPIRE-PHC to develop OHT data included: Client Agency Program Enrolment [CAPE], Community Health Centre [CHC], Ontario Health Insurance Plan [OHIP], National Ambulatory Record System [NACRS], Discharge Abstract Database [DAD], and Registered Persons Database [RPDB]. We also incorporated other administrative data from the 2021 Census of Population for the Peel census division [where Mississauga is located] to obtain additional contextual information, including marital status, family characteristics, and household and dwelling characteristics. We focused on data from the Mississauga OHT population as a sample population to make preliminary personas for only one population segment, frailty, to refine our persona methodology. This focus was chosen to evaluate the process and format of the personas instead of focusing on generating a variety of personas for multiple segments and OHTs, which will be done in future work and would be done in practice when taking a people-centred segmentation approach [as depicted in Figure 1].

INSPIRE-PHC data summarise the population for each OHT by demographic variables [e.g., age, sex, income], service user characteristics [e.g., levels of material deprivation, chronic illness], primary care indicators [e.g., number of primary care visits, follow-up care receipt], and healthcare utilisation measures [e.g., ED visits, hospital readmissions]. Each OHT’s data table from INSPIRE-PHC’s website displays these characteristics for the population overall and non-mutually exclusive service user sub-populations, defined as those who had ED visits or hospitalisations in the past two years, frailty issues, long-term home care, palliative care, and mental health issues. INSPIRE-PHC defined frailty using the Johns Hopkins Adjusted Clinical Groups [ACG] frailty-defining diagnoses indicator [37]. The ACG methodology for defining frailty includes 10 validated clusters of frailty diagnoses [i.e., malnutrition, dementia, impaired vision, decubitus ulcer, incontinence of urine, loss of weight, poverty, barriers to access to care, difficulty in walking, and falls].

#### Persona Formation

While there is no standard way to create personas, they should reflect the research objectives and target audience [16,17]. Grouping individuals by their primary disease or diagnosis is insufficient; instead, individuals should be clustered into cohorts with those sharing high degrees of similarity across clinical, personal, and behavioural traits to ensure a holistic understanding [13]. Typically, one to four personas are recommended per cluster to balance volume with practicality [16], and each should not exceed two pages. Information could include behaviour patterns, goals, skills, attitudes, background information, and the persona’s environment [38].

**Figure 2:**
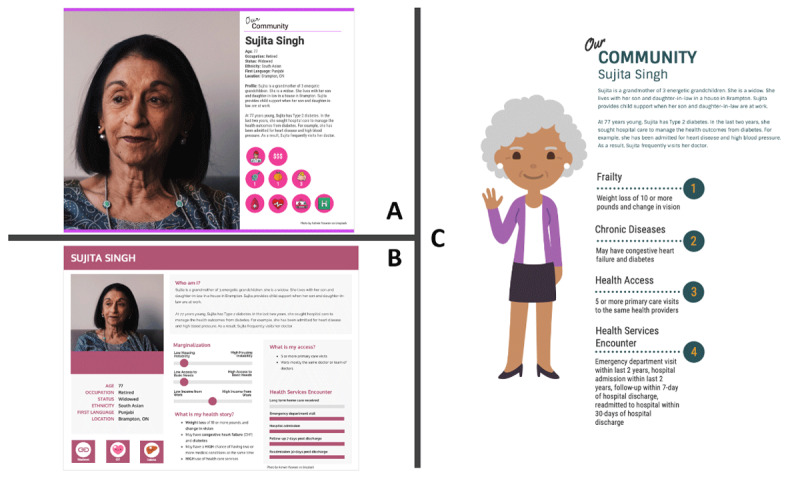
Focus group round 1 persona formats [A-icon-oriented, B-data-oriented, and C-highlights]. Personas were created Vengage*©* [38] and stock images from Unsplash*©* [39].

**Figure 3:**
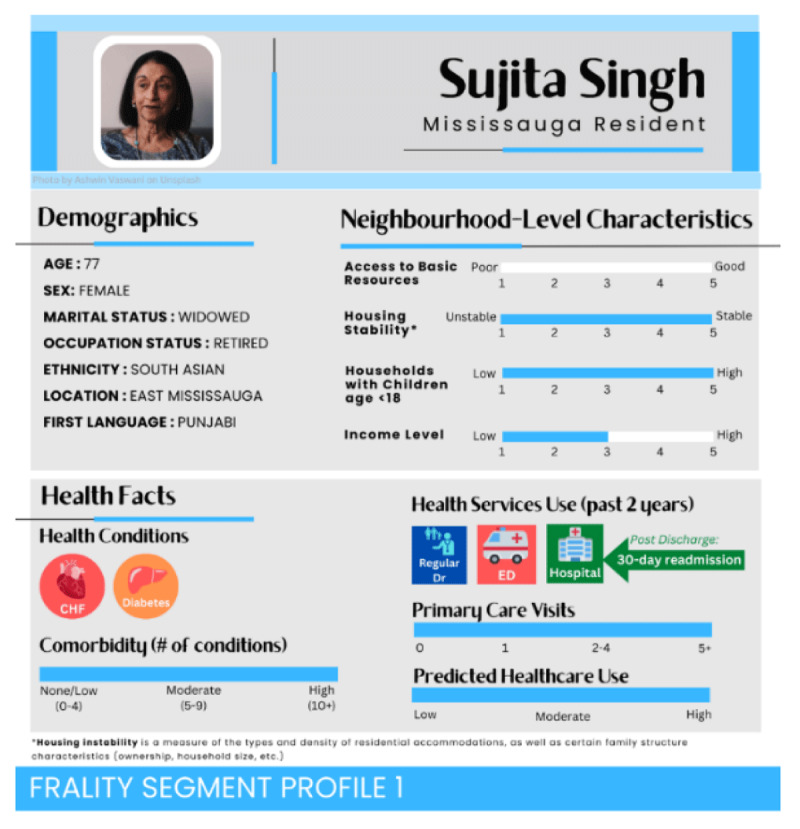
Persona in focus group round two. Personas were created on Canva*©* [41] and stock images from Unsplash*©* [40].

Given the breadth and diversity of the frailty population, six personas were developed for pilot testing [see **Appendix A** for an overview]. As per the INSPIRE PHC data reports for the Mississauga OHT, select characteristics were assigned to one of the six personas. Characteristics were assigned based on what was frequently reported in the data, which is a common method [39]. Using the data reports, the following assumptions were made for the frailty segment in Mississauga: 1] even distribution of males [51.6%] and females [45.4%]; and 2] most individuals self-identifying as coming from the Asia & Pacific [55.6%] world region. Frailty was defined as those with specific diagnoses; however, proportions of each specific diagnosis were not available in the dataset. As such, frailty-related diagnoses were arbitrarily assigned to each persona. Three visual formats were created for each of the six personas, including two photograph-based personas [icon-oriented – A, and data-oriented – B] and one with an avatar supplemented by informational highlights – C (Figure 2). A textual summary accompanying each format provided a high-level overview of each persona. Based on participant feedback from the first round of focus groups and their preference for options A and B, a second set of personas that hybridised between the icon and data-oriented personas were developed (Figure 3).

### Focus Groups

#### Recruitment

Working closely with our project team, we recruited a convenience sample of participants [service user, caregivers, and providers] that reflected or provided care to the chosen frailty population segment from Mississauga [round 1] and Ontario-wide [round 2]. Recruitment flyers specifically asking for people with frailty-related conditions and their caregivers or providers [not only physicians] were shared through our study team’s networks [e.g., patient and caregiver advisors, health system advisory/collaborative networks] via email and social media. We asked participants who expressed interest to fill out a screening questionnaire on Google Forms to ensure eligibility [ages 18 and over and residing in Mississauga for round 1 or Ontario for round 2] and their relevant experience related to the frailty segment [i.e., experiencing frailty themselves or providing care to those with frailty]. We also offered all participants a 25 CAD gift card as a token of appreciation for a 1.5-hour focus group.

#### Focus Group Format

We conducted two iterative rounds of virtual focus groups on Zoom, where the first round tested a broad range of persona formats derived from consulting existing resources, and the second round refined it. In each round, we conducted two separate focus groups, one with service users and caregivers and another with care providers [to ensure participant comfort and more opportunities for sharing]. Each focus group had three to four study team members, including two co-facilitators, one technical facilitator [to help with PowerPoint and technical issues], and one observer. All researchers took field notes on poignant discussion points or process-oriented impressions and gathered to debrief after each focus group. Data from the first round of focus groups were analysed, and results were summarised and used to inform and further refine the second round of focus groups.

**Figure 4:**
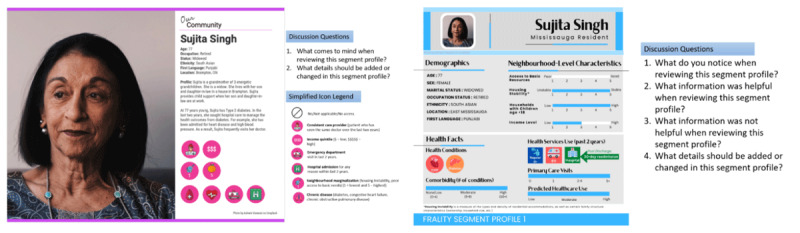
Slides for focus group rounds one [left] and two [right].

In the first focus group round, we displayed icon-oriented personas [format A in Figure 2] on PowerPoint slides that included discussion questions. The slides from the first focus group round contained a legend describing each icon. The second set of personas for the second focus group round did not require a legend describing each icon because icons [if any] had descriptors within them (Figure 4). The facilitator summarised what information was presented on each persona and led a discussion about what information resonated with the segment and what was lacking [related to background characteristics, needs, experiences, and social factors], to align with core elements of patient complexity, as outlined in a framework by Schaink et al. [41]. Participants were also asked to provide feedback on the format of the personas presented. At the end of the first focus group round, we presented all three persona formats and asked participants which they preferred and why. To ensure enough time for a rich discussion, we presented three personas that demonstrated a variety of individuals with frailty in both focus group rounds.

### Qualitative Analysis

All focus group discussions were audio-recorded and transcribed verbatim. Transcripts were analysed using NVivo 12 and with a directed content analysis approach using the aforementioned complexity framework [41] as a guide to help organise our focus group findings into five key categories: health and social experiences and needs, demographics, mental health, social capital, and physical health/disease characteristics. In addition to our directed coding approach using the complexity framework, we also applied an inductive and latent thematic analysis approach to derive insight into the process of turning segments into personas. These different angles of coding were separated to ensure the process coding only focused on the methodology and strategies used in the focus group and was not mixed in with the content related to personas.

A team of three qualitative analysts conducted the coding. Two coders analysed the content [coder 1 and 2; CC and JB], and one coder [coder 3; AB] focused on latent process coding, and all coders discussed progress and debriefed decisions at each step. The analytical method for both focus group rounds was the same, and separate codebooks were developed for each round to thoroughly reassess the methodology.

For content-coding, we drew mainly from Assarroudi et al.’s [42] process for directed content analysis, beginning with the creation of a categorisation matrix with the names and definitions of main categories and sub-categories. For this first step, we noted the five categories of the complexity framework as the main categories and used the framework’s examples for each category as sub-categories. With this initial matrix, coder 1 broadly applied the main categories to the text. This text was reviewed by coder 2, and both coders discussed any discrepancies in category assignment. Then, coder 1 went through the text for each main category and began describing transcripts using codes at the lowest level of abstraction [closest to the text]. Coder 1 grouped and organised these low-level codes and categorised these groupings under sub-categories from the complexity framework. Where these codes did not belong, coder 1 sorted them into an “other” sub-category. Coder 1 then finalised the codebook and transcripts, which were then reviewed by coder 2. Then, all three coders met to discuss and finalise the content codebook. Lastly, all coders reviewed codes and their associated transcripts to derive themes. This process involved examining where different sub-categories had overlapping codes.

For latent process coding, coder 3 reviewed the transcripts and applied inductive codes related to the process of running the groups and how participants received and engaged with the content. These process codes captured points of confusion, misinterpretation, or the need for further clarification by participants. For example, in the first round, participants asked for clarification on the purpose and use of personas for healthcare providers to better orient themselves in the discussion, leading us to provide a much more fulsome description of the use of personas in the next focus group round. Inductive codes were organised into themes centred on format, presentation, meaning, and facilitation. Once the process-oriented codebook was complete, the coding team met to discuss findings and how we would modify the focus group process based on learnings. In both cases, coders reviewed field notes in the final coding meetings to ensure initial impressions during the focus groups were represented in the codebooks.

### Ethics

Research ethics approval was granted by the Trillium Health Partners Research Ethics Board on June 1, 2023 [ID#1099].

## Results

Between both focus group rounds, we recruited 27 participants, with 3 service users, 12 caregivers [some of whom were also service users], and 12 providers [including physicians, allied health professionals, and social care providers].

### Process Learnings

#### Persona Design

Overall, focus group participants preferred the more data-oriented format [format B in Figure 2] of the persona, with the main reason being that sliding scales are easier to interpret and more intuitive than icons with numbers.

Information presented in the personas that all participants found helpful in the first round of focus groups included demographic information [especially age, income, city of residence, marital status, and language spoken], chronic conditions, healthcare utilisation, long-term care residence, and access to basic needs [marginalisation]. Providers from the first focus group round uniquely found usual primary care provider availability helpful. In the second round of focus groups, helpful information included employment status, first language, and income. Service users and caregivers in the second round of focus groups thought it was helpful to know about an individual’s medical conditions and providers liked knowing whether an individual sees a regular physician. **Appendix B** contains a table outlining all information discussed by participants and whether they are available in health or other administrative datasets.

Participants from the first round of focus groups were confused by neighbourhood-level marginalisation measures and asked why these measures were chosen and how they were to be applied at the individual level, especially if the neighbourhood characteristics were not immediately consistent with individual characteristics [e.g., high neighbourhood housing stability and low household income]. Another source of confusion was that each neighbourhood-level measure’s number [1,2,3,4,5] was difficult to interpret because each measure’s title was negatively named; yet, lower values represented more positive conditions [e.g., for housing instability, 1 was favourable and 5 was unfavourable]. Lastly, some participants expressed that it was difficult to understand specific income amounts since only arbitrary levels [1,2,3,4,5] were shown. Given these comments, we created a data-focused persona for our second round of focus groups [Figure 3] with sliding scales and fewer icons. We eliminated the textual summaries because we recognised that we wanted to understand how participants reacted to the data on their own and test what necessary information could not be gathered from data if we gave the maximum variety of information that could be gathered from data. In the second round, we explicitly titled and featured neighbourhood-level measures at the top of the persona to improve clarity from the first round. However, while participants wanted to know these measures at the individual level, they reported that these measures should be less emphasised at the neighbourhood level and serve as supplementary information only. Additionally, with neighbourhood-level characteristics at the top of the infographic, participants were unsure if this emphasis made sense given health systems planning is being done at the segment level rather than the neighbourhood level.

#### Focus Group Facilitation

It was not clear to participants how and to what degree the personas represent a segment and how the personas will eventually be used. They wanted a clearer framing of how health system decision-makers would be using them. We clarified the script in the second round of focus groups to ensure this was understood [see **Appendix C**].

### Content Themes

We identified 7 themes based on what all participants said they wanted to know about each frailty segment persona: the person’s social networks, interactions, support, communication abilities, self-management and independence, attitude towards condition, priority of diagnoses, mental health impact on frailty, and physical context. These themes are briefly summarised below.

**Social networks, interactions and support** involve an individual’s social life, including social activities, formal or informal relationships, and, notably, relationships with family. A caregiver noted wanting to know about familial responsibilities and community ties:

“I just wondered about her social activities from a support network perspective. You know it’s a lot to – you know she’s looking after small children and she’s looking after […]. Are there other people even from a perspective that she can gain other support from, whether it be her religious community, her – you know maybe it’s her bridge group or card group, her reading club?” – Caregiver 1

A service user also noted the importance of an individual’s family dynamics and how they may affect the individual’s level of support:

“[…] it’s a huge shock that she’s the one providing care to the grandkids, to the daughter in law, to the son […][she] was the caregiver and now she’s the patient herself, so just having those resources so she understands that complexity and what is out there for her so she doesn’t feel alone […] a lot of our parents for that generation, they feel like a burden to the family that they’re useless, all they do is just sleep and eat, especially in a cultural perspective, so letting them know that you’re not a burden. The family is here to support you.” – Service User 1

A provider extended these ideas in the sense of wanting to know about the health of an individual’s partner and their ability to support one another:

“[what] I would want is for his partner’s physical condition. […] how healthy [is] his partner and did he [need] to take care of her, or can they support each other, or they need others like relationships with his kids. Because it said he lived with his kids in this house, I think maybe […] his kids still live with them or not.” – Provider 1

An individual’s **ability to communicate their needs and priorities or understand information** was also critical, in terms of comfort with languages and understanding of medical terms but also hearing impairment, which a caregiver described:

“[…] regarding the language part […] someone earlier mentioned that as well, about whether the person is able to manage stressful situations like going to the doctor, going to the hospital on their own. Based on my experience with my mother who speaks English quite well, however, because of her hearing – she’s going to be 85 soon – because of her hearing […] she gets stressed in the doctor’s office” – Caregiver 2

**Self-management and independence** were discussed heavily, which refer to individuals’ ability to be independent and care for themselves, both in maintaining their health and accessing healthcare and other services. A provider described what this entails:

“I know it doesn’t state a lot about his independence so exploring that, as well as the partner’s independence, as well when it comes to the regular ADLs [activities of daily living] or whether they’re able to cook fresh food for themselves, you know, especially given his diabetes and that he has seen his weight change. So what kind of food is he consuming, is it store-bought, fast food.” – Provider 2

**Attitudes towards condition** was highlighted by a participant when they explained the difference in attitudes between their parents regarding frailty:

“My dad was frail, but he was mad about it. And he pushed himself to do things and wouldn’t listen to people if we said, you’ve got to be careful, you’ve got to do this […] he was perfectly fine using a walker. Whereas my mom being frail, she’s so afraid that she’s going to fall again that she holds herself back […] she completely resents the walker and wishes she didn’t have to use it.” – Caregiver 3

The **priority of diagnoses** was also noted as an important consideration by a caregiver, who is also a service user, when they described their multiple frailty diagnoses:

“[…] I would say at this point in my life, my colitis is probably more limiting than any other of my diagnoses, even though everybody thinks heart failure is probably the worst thing that can happen to you. […] If somebody asked me what I most wish I could improve right now, if they could take one of my conditions away from me, which one would it be, I would pick the colitis.” – Service User/Caregiver 1

A participant also mentioned the importance of the **mental health impact on frailty**:

“Also, in terms of comorbidity, mental health issues, both directly related to the frailty. And in my mother’s case and from what I’m understanding more and more with taking care of her is, really, it applies to a lot of seniors who are in a situation like my mother, that the mental health issues, depression, just underlies the lives of most of them and exacerbates whatever physical condition they’re in.” – Caregiver 2

**Physical living context** refers to an individual’s physical environment, including whether they have access to housing or other necessities, their place of residence, and people or family in their environment. A provider listed the vast array of contextual characteristics that would be helpful to know:

“[…] multigenerational homes, how many folks live in the home, that informal support sort of could be identified. I mean here it appears that it’s just his partner and him, but we don’t know whether or not the adult children are there or, you know, if they’ve taken in a boarder.” – Provider 3

A provider also noted the importance of housing affordability and stability, as it is a concern in their day-to-day role as a physician:

“What’s more important for the most part, is precarious housing and how much of your income is actually going for rent. And it’s sort of more than 50 per cent income going to rent, is a standard definition, federally of what core housing need and housing stability is. But that’s only fed financial housing stability, but also other dimensions of housing stability.” – Provider 4

## Discussion

We demonstrated and assessed a novel process for developing personas for population segmentation. This approach allowed us to incorporate community perspectives into better understanding and using population segments by creating personas. We found that summarising available health and other administrative data on individuals from a segment can provide a basic understanding of a segment. However, these data were insufficient as our participants were consistently inclined to dig deeper and understand information that cannot be gleaned from health or other administrative data, which were captured by our focus group findings and themes [see **Appendix B**]. For example, even though we may know people’s diagnoses from the data, participants identified the importance of knowing how people prioritise different conditions. Thus, it is evident that qualitative data is necessary to obtain specific information on the service user perspectives, needs, and priorities that may be necessary for planning. We also note several novel learnings in effectively using personas to inform population segmentation. Specifically, neighbourhood-level characteristics related to the social determinants of health can be helpful, but participants still desired to know what these were at the individual level. Moreover, people-centred population segmentation is conceptually challenging for participants to understand and required us to dedicate more time to discuss further and do context-setting before proceeding with focus groups.

As seen in Parkland Health’s segmentation exercise [13] and many other health systems’ segmentation initiatives [11,12], population segmentation has great potential to directly support planning health services and programs around people within each segment. However, similar to our findings, many have noted the importance of incorporating holistic data that captures service user perspectives and the social determinants of health [43]. This is in line with a study by Yoon et al. [44] that interviewed stakeholders from the government, researchers, and care providers about what the optimal indicators were for population segmentation and what purpose it should serve. Stakeholders noted that health and sociodemographic information was important, as well as behavioural characteristics, functional status, and psychosocial factors. They also noted that population segmentation should be person-centric and incorporate upstream and holistic indicators to support resource allocation appropriately. An additional implication of our findings is that the inputs provided by participants highlight specific social, contextual, and needs-related information that are currently absent from routinely collected health administrative data in Ontario. By surfacing these gaps, the use of personas can inform opportunities to strengthen existing data infrastructure through the inclusion of new data elements that better capture the lived experiences and determinants of health that are critical for population segmentation. Addressing these gaps could ultimately enhance the discriminatory power and relevance of data-driven segments for population health management at scale.

We discovered a trade-off from using personas where we benefit from participants being able to anchor to an individual representing real people from a segment but may lose some applicability at the health system level because results are highly person-specific. Even when we clearly described in the second focus group round that this discussion was meant to inform health systems’ decision-making [versus individual clinical care], participants still discussed each persona as if they were representing individuals and used to inform individual care. A solution would potentially be to indicate the proportion of individuals from the population that each persona represents or implement personas that refer to a group of individuals [i.e., no picture, specific name, and age], which some studies have adopted for segmentation [45]. This format may broaden the scope of responses from participants for results to be more applicable for population health management and planning around segments.

### Limitations

There are several limitations in our suggested persona approach and our study itself.

Pertaining to our approach, we ensured that participants understood the concept of population segmentation and its ability to support system-level decisions [see **Appendix C**]; however, we did not formally standardise participants to a uniform definition of population segmentation in the context of population health management. As such, the personas generated from our approach cannot provide statistical discriminatory power or outcome validation and should be viewed as complementary, rather than as a replacement for data-driven approaches and ongoing studies to validate data-driven population segmentation for population health management. In line with this view, we focused on eliciting a broad range of perspectives on how we can meaningfully represent an individual from a segment instead of forming discriminatory segments. Additionally, our approach relies on a careful recruitment strategy. It will likely be the case that entities taking this approach will not be able to contact individuals directly from a segment, since data are anonymised. In our case, we could not contact and recruit the exact people from Mississauga’s frailty sub-population from the data since data were anonymised. Instead, we recruited people from Mississauga via our networks who could relate to the segment [e.g., frail adults, their caregivers, or providers who care for them].

Specific to our study, we had challenges recruiting frail service users; thus, our data may not capture their perspectives. We addressed this by recruiting broadly from Ontario in the second focus group round and were able to obtain more service users; however, future work should consider more tailored ways of recruiting individuals from segments [e.g., recruiting in person]. Our study also used existing sub-populations as segments and only focused on three types of individuals within the frailty population. The data used to create these segments also did not contain comprehensive social data. This limited our ability to evaluate the complete process of people-centred population segmentation, including a comprehensive segmentation strategy. However, we argue that our focus on incorporating qualitative methodologies allowed us to refine persona development from population segments. Our findings support expanding population segmentation based only on administrative data, as it may be limited, by engaging communities in persona development. Lastly, given the focus on assessing the persona methodology, the results of our study should not be used to finalise or generalise all themes about the frailty population in Mississauga. It serves as an important starting point for service user and provider concepts that require further study for population segmentation. In future work, we will explore and evaluate a more comprehensive population segmentation methodology using available data on health system users in Ontario.

## Conclusion

Our study demonstrates that a community-engaged population segmentation approach involving the creation of personas can enable the integration of health and social needs and priorities of sub-groups within a population. The breadth of information captured through our proposed methodology can feed into a population health management approach that embraces diverse needs and draws on an integrated network of services [health, community, and social services]. Future work will involve further testing and refining this approach over various segments and using personas to design integrated care pathways.

## Additional File

The additional file for this article can be found as follows:

10.5334/ijic.9002.s1 Appendixces. Appendix A to C.
