# Supplementary material for: Using Personas as a Tool for People-Centred Segmentation and Health Systems Planning in Ontario, Canada
Source: Int J Integr Care. 2026 May 21;26(2):12. doi: 10.5334/ijic.9002 (PMC13196677; doi:10.5334/ijic.9002)
Supplement: Appendixces. — Appendix A to C. [file ijic-26-2-9002-s1.pdf]

Appendix A – Overview of Six Personas [*indicates the personas used in focus groups; round 2 used profile 6 instead of 4]

CATEGORY	SEGMENT PROFILE 1	SEGMENT PROFILE 2*	SEGMENT PROFILE 3*	SEGMENT PROFILE 4*	SEGMENT PROFILE 5	SEGMENT PROFILE 6*
**Name**	Ameena Padhi	Sujita Singh	Anil Manji	Hao Wu	Crystal [Xia Lin] Li	Charles Jefferson
**Frailty**[1]	Diagnosed with early onset dementia	Weight loss of 10 or more pounds and change in vision	Has difficulty walking	Weight loss of 10 or more pounds	Falls from time to time and has bedsores	Lack control of urine
**Person**						
Age	82	77	80	71	65	64
Sex	F	F	M	M	F	M
Attachment	Attached	Attached	Attached	Attached	Uncertainly Attached	Attached
Income Quintile	4	3	4	3	2	5
Ethnic Con.	5	5	5	5	5	5
Marg[2]: Instability	5	1	4	2	5	5
Marg[2]: Deprivation	1	1	3	2	3	2
Marg[2]: Dependence	2	3	2	1	1	2
Marg[2]: Ethnic Con	5	5	5	5	5	5
Recent Imm[3].	No	No	No	No	No	No
Comorbidity	High	High	High	High	Moderate [Limited]	High
Morbidity	High	High	Moderate	High	High	High
Chronic Disease	No	Diabetes CHF	No	Diabetes	COPD	CHF
**Place**						
RIO Index	Urban	Urban	Urban	Urban	Urban	Urban

[1] Frailty was defined in INSPIRE PHC data using the Johns Hopkins Adjusted Clinical Groups [ACG] frailty-defining diagnoses indicator. It is based on 10 clusters of frailty-defining diagnoses [i.e., malnutrition, dementia, impaired vision, decubitus ulcer, incontinence of urine, loss of weight, poverty, barriers to access to care, difficulty in walking, and falls]. The ACG frailty indicator captures patients with multidimensional frailty at the population level and has been shown to accurately identify patients with limitations in activities of daily living.

[2] Marg = Marginalisation; measured in quintiles and based on the Ontario Marginalisation Index [ON-MARG] – a geographically [Census] based index developed to quantify the degree of marginalisation occurring across the province of Ontario. It is comprised of 4 major dimensions thought to underlie the construct of marginalisation: residential instability, material deprivation, dependency and ethnic concentration.

[3] Imm. = Immigration

CATEGORY	SEGMENT PROFILE 1	SEGMENT PROFILE 2*	SEGMENT PROFILE 3*	SEGMENT PROFILE 4*	SEGMENT PROFILE 5	SEGMENT PROFILE 6*
LTH[4] received	No	No	Yes	No	No	No
Enrolment model	CAP	CAP	CAP	FHT	NOG	CAP
Core PC[5] visits	5+	5+	5+	5+	5+	5+
3+ physician	Rostered	Rostered	Rostered	None	Not Rostered	Rostered
3+ own group	Rostered	Rostered	Rostered	None	Not Rostered	Rostered
ACSC[6] hosp.	No	No	No	No	No	No
**Time**						
Palliative care	No	No	No	No	No	No
MH[7] illness	Yes	No	No	No	No	No
ED[8] visit	Yes	Yes	Yes	No	Yes	Yes
Urgent ED[8]	Yes	No	Yes	No	Yes	Yes
Non-urgent ED[8]	No	No	Yes	No	No	No
Hosp. admit.	Yes	Yes	Yes	No	Yes	Yes
7-day follow-up	Yes	Yes	Yes	Yes	Yes	Yes
30-day re-admit	No	Yes	No	No	No	No

[4] LTH = Long term homecare
[5] PC = primary care
[6] ACSC = ambulatory care sensitive condition
[7] MH = mental health
[8] ED = emergency department

Appendix B – Important Information Available/Unavailable in Health and Other Administrative Data [discussed by participants; mapped to the 5 complexity framework dimensions]

Complexity Framework	Information Available in Administrative Data	Information Unavailable in Administrative Data
**Health and social experiences**	- Healthcare utilisation [inpatient/outpatient, emergency department, long-term care, home care, palliative care, re-admissions, follow-ups]	- Health system navigation<br>- Self-management and independence<br>- Community service access<br>- Caregiver availability<br>- Health education and communication abilities<br>- Food security and access<br>- Caregiver access and characteristics<br>- Caregiver status [are they a caregiver?]<br>- Housing access<br>- Living conditions<br>- Health/social service affordability and availability<br>- Medication management<br>- Quality of life
**Medical and physical health**	- Healthcare utilisation [inpatient/outpatient, emergency department, long-term care, home care, palliative care, re-admissions, follow-ups]<br>- Medical/health diagnoses<br>- Prescription drugs	- Physical functioning and mobility<br>- Diet and nutrition<br>- Long-term healthcare needs with decline<br>- Alternative medicine use<br>- Health behaviours and lifestyle factors
**Social capital**	- Neighbourhood marginalisation characteristics [indicator of socioeconomic status]	- Social support and relationships [family, formal, and informal]<br>- Activities<br>- Knowledge about resources
**Demographics**	- Sex<br>- Age<br>- Immigrant status and world region of origin<br>- Geographical area of residence and rurality<br>- Primary care continuity [number of visits to physician]	- Culture, race, and ethnicity<br>- Language understood/spoken<br>- Employment or occupation [current/past]<br>- Religion<br>- Digital literacy<br>- Individual/household income<br>- Marital status<br>- Educational attainment<br>- English as a first language
**Mental health**	- Mental health and additions-related diagnoses and healthcare utilisation	- Cognitive capacity<br>- Psychological wellbeing

__Focus Group Round 1 Script:__ ***"The purpose of this focus group today is to share with you what we have learned about the characteristics of people who have a healthcare issues related to frailty in Mississauga and surrounding regions. We created profiles of population segment using health care data from this region through an exercise called population segmentation. Since this process is solely based on data, we know that we are missing important information, which is why we have gathered today. We are going to review our segment profiles [which are data-based stories of individuals in the frailty segment] and get your feedback on how we can make these profiles better reflect the characteristics, needs and experiences of these individuals in your community."***

__Focus Group Round 2 Script:__ ***"Population segmentation can broadly be defined as taking an entire population and dividing them into groups based on shared and similar health-related characteristics and needs. The purpose of this focus group today is to share with you what we have learned about the segment or group of people who have healthcare issues related to frailty, generated from population segmentation. In forming this group, frailty was defined as having frailty-defining diagnoses such as malnutrition, dementia, as well as poverty or other barriers to access to care. Many individuals within this group also usually have limitations in activities of daily living.***

I will give a brief summary of what population segmentation is but later we will go into more detail about the process. We segmented the frail population and created profiles using available health care data from the Mississauga region as an example region. Since this process is solely based on data, we know that we are missing important information, which is why we have gathered today. We are going to review our segment profiles [which are data-based stories of individuals in the frailty segment and presented today as infographics] and get your feedback on how we can make these profiles better reflect the characteristics, needs and experiences of these individuals in your community. For a lot of characteristics, we only have area-based measures instead of individual-level characteristics. We also hosted focus groups with patients and caregivers in Ontario and their feedback will also be incorporated into the results.

Eventually, results from these focus groups will inform how Ontario Health Teams in the province can make best use of population segmentation. We envision that Ontario Health Teams will segment the population they serve, understand each population segments' characteristics, and use them to plan services and supports for the entire frailty segment."

[housekeeping script about focus group length and instructions]

Right before segment personas are shown: *"As previously mentioned, we used a method called population segmentation which involved using available health data to create distinct profiles of individuals who have health conditions related to frailty. This was done by first, identifying a full population's data and pulling available characteristics from the data to learn about individuals in the population. After understanding these characteristics of the population, we segmented or grouped individuals with similar characteristics together. The main goal of this project is to do people-centred population segmentation, which is shown in step 3. People-centred population segmentation involves generating personal stories meant to represent individuals in the segment. This is done using the same data that's used to understand population characteristics, but also community input, through these focus groups. Ultimately, the goal of this entire process is to help health systems understand groups in the population to inform planning and care provision.*

As a first step to bringing the segment to life through stories, we created generic profiles to capture the diversity of individuals in the frailty segment in the city of Mississauga as an example and we will show you three of them today to get your feedback. Please note, these are not real people from the frailty segment but are representative of people you might find in it. They were generated from the frailty segment to facilitate our discussion. The ultimate goal to keep in mind is that these profiles are being used to bring a segment of the population from data to life for health systems decision-makers to enable planning and decision-making for frail individuals at the systems level."
